# SOCIAL COGNITIVE DEFICITS RELATE TO KEY ASPECTS OF OLDER ADULTS' SOCIAL NETWORKS

**DOI:** 10.1093/geroni/igac059.953

**Published:** 2022-12-20

**Authors:** Anne Krendl, Brea Perry

**Affiliations:** Indiana University, Bloomington, Indiana, United States; Indiana University, Bloomington, Indiana, United States

## Abstract

Social connectedness confers benefits to older adults’ cognition, including slowing the progression of Alzheimer’s disease (AD). Social connectedness is facilitated by social cognitive function – how people understand, store, and apply information about others – which declines over the lifespan. We examined whether two core social cognitive skills – face memory and theory of mind (the ability to infer others’ mental states) – predicted older adults’ social network structure and composition. Cognitively normal older adults (OA; N=119) and OA with mild cognitive impairment (MCI) or AD (N=96) completed a social network interview, a face memory task, and a theory of mind measure. Social cognitive deficits were highest among OA with MCI and AD. Face memory predicted network size, whereas theory of mind predicted network composition. Neuroimaging results describing OA’s social cognitive deficits are also discussed. Social cognitive function may be an important intervention target for preserving older adults’ social connectedness.

